# Supplementary material for: Splice-Junction-Based Mapping of Alternative Isoforms in the Human Proteome
Source: Cell Rep. Author manuscript; Available in PMC 2020 Jan 15. (PMC6961840; doi:10.1016/j.celrep.2019.11.026)
Supplement: 2 [file NIHMS1546469-supplement-2.zip › DF1/PXD006675/RightAtrium/RightAtrium_51_NRAP_ANAANLSEVK.pdf]

A

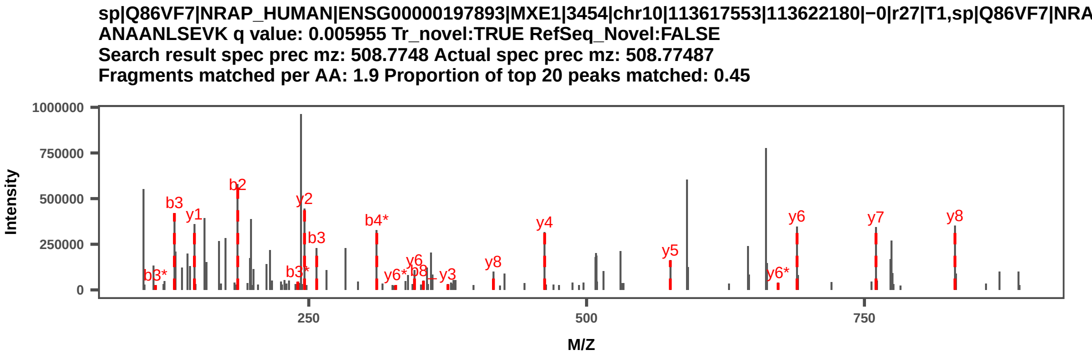
sp|Q86VF7|NRAP_HUMAN|ENSG00000197893|MXE1|3454|chr10|113617553|113622180|−0|r27|T1,sp|Q86VF7|NRA
ANAANLSEVK q value: 0.005955 Tr_novel:TRUE RefSeq_Novel:FALSE
Search result spec prec mz: 508.7748 Actual spec prec mz: 508.77487
Fragments matched per AA: 1.9 Proportion of top 20 peaks matched: 0.45
Intensity
M/Z
1000000
750000
500000
250000
0
250
500
750

B

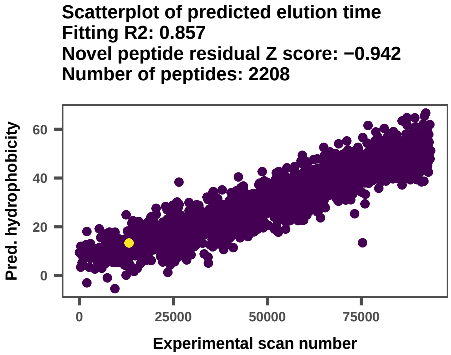
Scatterplot of predicted elution time
Fitting R2: 0.857
Novel peptide residual Z score: −0.942
Number of peptides: 2208
Pred. hydrophobicity
Experimental scan number
0
20
40
60
25000
50000
75000

C

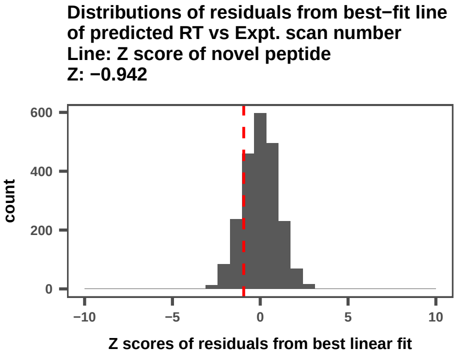
Distributions of residuals from best−fit line
of predicted RT vs Expt. scan number
Line: Z score of novel peptide
Z: −0.942
count
Z scores of residuals from best linear fit
0
200
400
600
−10
−5
0
5
10
